# Mathematically modeling fluid flow and fluid shear stress in the canaliculi of a loaded osteon

**DOI:** 10.1186/s12938-016-0267-x

**Published:** 2016-12-28

**Authors:** Xiaogang Wu, Ningning Wang, Zhaowei Wang, Weilun Yu, Yanqin Wang, Yuan Guo, Weiyi Chen

**Affiliations:** Shanxi Key Lab. of Material Strength & Structural Impact and College of Mechanics, Taiyuan University of Technology, Taiyuan, 030024 People’s Republic of China

**Keywords:** Canaliculi, Fluid flow rates (FFR), Fluid shear stress (FSS), Poroelasticity, Osteon

## Abstract

**Background:**

Mechanical load-induced intraosseous pressure gradients may result in some fluid stimuli effects, such as fluid flow and fluid shear stress (FSS), which may enable bone cells to detect external mechanical signals. Interstitial bone fluid flow is known to occur in lacunar–canalicular porosity (PLC).

**Methods:**

In order to characterize lacunar–canalicular fluid flow behavior, a hierarchical osteon system is developed. The osteon is modeled as a poroelastic annular cylinder with two types of impermeable boundary cases considered on its outer wall: one is elastic restrained (Case I), whereas the other is displacement confined (Case II). Analytical solutions such as canalicular fluid velocity, pressure, fluid flow rate (FFR), and shear stress are obtained.

**Results:**

Results show that the amplitudes of FFR and FSS are proportional to strain amplitude and frequency. However, the key loading factor governing canalicular fluid flow behavior is the strain rate. The larger canalicular radius is, the larger amplitudes of FFR and FSS generalized, especially, the FSS amplitude is proportional to canalicular radius. In addition, both FFR and FSS amplitudes produced in case II are larger than those of case I.

**Conclusion:**

Strain rate can be acted as a representative loading parameter governing the canalicular fluid flow behavior under a physiological state. This model can facilitate better understanding the load induced the fluid permeation in the PLC. The approach can also be used to analyze the structure of the proteoglycan matrix in the fluid space surrounding the osteocytic process in the canaliculus.

## Background

As a poroelastic material, bone often bears cyclic loads that might come from walking, running, or other daily activities [1]. These activities enable bones to “feel” and “adapt”, this is the mechanism called bone mechanotransduction. Intraosseous pressure gradients may result in some fluid stimuli effects (fluid flow, fluid shear stress (FSS), and streaming potentials), which may enable bone cells to initiate the process of adapting bone mass and structure to the environment [2]. This process may be the basic mechanism of bone mechanotransduction. Although the constituents and ultrastructure of bone interstitial fluid pathways remain poorly understood because of technical difficulties, interstitial fluid flow in bone tissue has been suggested to serve an important role in bone adaptation and metabolism [2].

Research for the intraosseous fluid stimuli should be conducted at the osteon level [1]. Osteon is the basic bone structure unit with a cylindrical structure, which is made of lamella around a Haversian canal. The Haversian canal contains blood vessel(s), nerve, and some spaces occupied by bone fluid. The wall of a Haversian canal is covered with cells, and behind the cells is the entrance to the bone canaliculi [3]—the passageways between lacunae and the Haversian canal. The space in the lacunae and the canaliculi is called lacunar–canalicular porosity (PLC). Mechanical load induced interstitial bone fluid flow is known to occur in this porosity [3–6]. This porosity also provides an ideal milieu for transfer of exogenous and endogenous signals via mechanical, electrical, and chemical mechanisms [7].

The PLC dimension is characterized by the radius of the canaliculus (order = 0.1 μm) [3]. The canaliculi have been regarded as the passageway that allows nutrients, oxygen, and wastes to be exchanged between blood vessels within the Haversian canal and osteocytes [8]. The canaliculus is the canal associated with slower relaxation of excess pore pressure and may also serve an important mechanosensory role [8] by providing the channels through which osteocytes can sense FSS [3, 4].

FSS plays an important role in modulating cell functions, and several laboratory systems have applied FSS to cells [9]. Theoretical models have been established to investigate intraosseous fluid flow behavior and FSS in homogeneous porous bone specimens [6, 10, 11] or in single osteon [12–17]. However, the osteon has different properties in longitudinal and radial directions, and the fluid flow in canaliculi is not directly coupled with mechanical loading [1]. Thus, a transverse isotropic poroelastic hollow osteon model [18–23] is developed to simulate osteonal poroelastic behavior. However, these theoretical models do not directly link the mechanical loading at the osteon scale to the scale of canalicular fluid flow.

Many theoretical models [6, 16, 24–26] have been established to deduce the canalicular fluid flow behavior, but its mechanism remains poorly understood because it is difficult for obtaining reliable experimental information in vivo at the nanoscale. Some authors have identified the canalicular fluid flow evaluated from Darcy’s law or Brinkman’s equation [6, 27]. To amend the above zero charge flux assumption, a highlighted model (by using the linearity of the Stokes–Brinkman differential equation) on the basis of an up-scaling approach has been proposed to quantify electroosmotic and hydro electrochemical effects [28–31]. Canalicular fluid flow associates with hydraulic, chemical, and electrical phenomena. Hydraulic flow and shear stress are generally the most relevant fluid stimuli effects induced by pressure differences in the interstitial bone fluid [32]. The aforementioned models are complicated and expatiatory, but those physical effects are not truly coupling in the motion equation of solutions (e.g., in Sansalone et al. [32] “Hydraulic, osmotic, and electroosmotic contributions to the fluid transport were split and treated independently using the linearity of the Stokes–Brinkman differential equation”). In other words, fluid motion equations might no longer work for those coupled physical effects. Chemical, osmotic, and electroosmotic phenomena are hysteresis band effects induced by canalicular fluid flow. The first physical effects of canalicular fluid flow behavior are fluid pressure gradient, velocity, and viscous force.

Therefore, we will establish a hierarchical model to directly evaluate the first physical effects of canalicular fluid transport behavior, which allows the linking of cyclic mechanical loading on osteon to the fluid flow in the canaliculi. The lacunar–canalicular primary porosity scale is proposed to relax the pore pressure associated with FSS because of mechanical loading. A homogenization canaliculated porosity approach may avoid multiscale parameters of permeability. We specifically develop a canaliculi model on the basis of our previous osteon model [33–36] to link the external loads to the canalicular fluid velocity, pressure, flow rates, and shear stress. The model can also be used to analyze the structure of the proteoglycan matrix in the fluid space surrounding the osteocytic process in the canaliculus.

## Methods

**Fig. 1:**
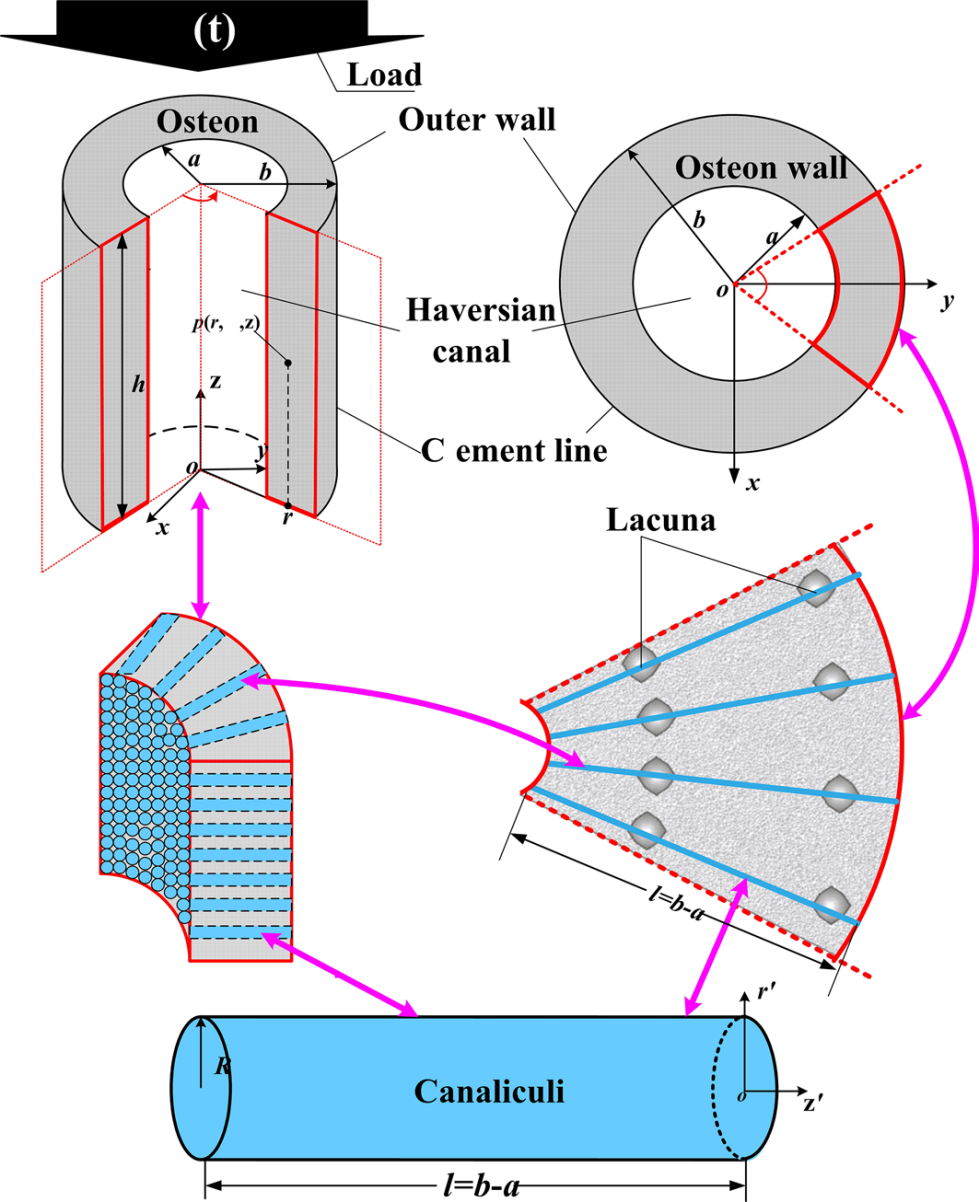
The Hierarchical model for osteon system. *a* and *b* are the inner (Haversian canal surface) and outer osteon radius, respectively, and *h* is height

**Fig. 2:**
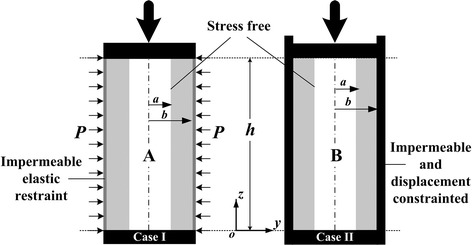
The osteon outer wall in case I and case II are assumed to be elastic restrained (**A**) and displacement confined (**B**) respectively

Biot’s poroelastic theory is used to account for fluid–solid interactions in osteon tissue. According to Wu et al. [36], the osteon system is modeled as a hierarchical hollow annular cylinder structure (as shown in Fig. 1) and two types of boundary cases for the osteon wall are proposed (as shown in Fig. 2). In this hierarchical structure, the canaliculi are assumed to be straight tubes and evenly distributed in the osteon wall, which run across the lamella and stop at the cement line. The viscous canalicular fluid flow and shear stress on the canalicular wall are induced by the fluid pressure difference and the effect of lacuna is neglected.

According to Wu et al. [36], the solutions of whole pore pressure distribution *p*_*m*_ = *p*_0*m*_(*r*)*e* ^*iωt*^ ($\omega$ is the angular frequency of loading, *m* = 1 is for case I and *m* = 2 is for case II) in the osteon wall is given by:

1 $$p_{m} = \frac{{MM_{11} \left( {\alpha c_{m} + \alpha^{\prime}\varepsilon_{z0} } \right)}}{{M_{11} + M\alpha^{2} }}\left[ {\frac{{I_{0} \left( {Cr} \right)K_{1} \left( {Cb} \right) + K_{0} \left( {Cr} \right)I_{1} \left( {Cb} \right)}}{{I_{0} \left( {Ca} \right)K_{1} \left( {Cb} \right) + I_{1} \left( {Cb} \right)K_{0} \left( {Ca} \right)}} - 1} \right]e^{i\omega t} ,$$

where, $M_{11} = E_{r} (E_{z} - E_{r} \mu_{z}^{2} )(1 + \mu_{r} )^{ - 1} (E_{z} - E_{z} \mu_{r} - 2E_{r} \mu_{z}^{2} )^{ - 1}$ is an elastic component of the stiffness tensor. *E*_*r*_ and *E*_*z*_ are radial and longitudinal Young modulus, *μ*_*r*_ and *μ*_*z*_ are radial and longitudinal drained Poisson’s ratio, *I*_*n*_ and *K*_*n*_ are the first kind and the second kind modified Bessel function of order *n* respectively, and *c*_*m*_ is constant and can be found in Ref. [33]. The constant C is given by Wu et al. [1, 33–36].

2 $$C = \sqrt {i\omega \mu \left( {M_{11} + M\alpha^{2} } \right)/\left( {kMM_{11} } \right)} .$$

where $i = \sqrt { - 1}$, *μ* and *k* are dynamic viscosity and intrinsic permeability, respectively.

According to the established canaliculi model in Wu et al. [36], the solutions for pressure *p′* and longitudinal velocity component *u* in the canaliculi are presented as:

3 $$p^{\prime}(z^{\prime}) = \left[ {p_{0} (a) + \frac{{p_{0} (b) - p_{0} (a)}}{l}\left( {z^{\prime} - a} \right)} \right]e^{i\omega t} ,$$

4 $$u(r^{\prime}) = \frac{{p_{0} (a) - p_{0} (b)}}{i\omega \rho l}\left[ {1 - \frac{{I_{0} \left( {\beta r^{\prime}} \right)}}{{I_{0} \left( {\beta R} \right)}}} \right]e^{i\omega t} .$$

where, *ρ* is the fluid density, and *μ* is the dynamic viscosity. The canalicular radius is *R* and lengthened *l* (*l* = *b* − *a*). The constant *β* is given by.

5 $$\beta = \sqrt {i\omega /\mu }$$

According to Weinbaum et al. [6] and You et al. [37], the fluid flow rate, *Q*, and shear stress, *τ*_*w*_, on the canalicular wall or osteocyte process membrane are given by:

6 $$Q = \int_{0}^{R} {2\pi r^{\prime}} u(r^{\prime})dr^{\prime},$$

7 $$\tau_{w} = \mu \left. {\frac{{\partial u(r^{\prime})}}{{\partial r^{\prime}}}} \right|_{{r^{\prime} = R}}.$$

Thus finally, we can obtain the FFR and FSS:

8 $$Q_{m} = \frac{2\pi }{i\omega \rho l}p_{0m} (b)\left[ {\frac{R}{\beta }\frac{{I_{1} \left( {\beta R} \right)}}{{I_{0} \left( {\beta R} \right)}} - \frac{{R^{2} }}{2}} \right]e^{i\omega t}$$

9 $$\tau_{wm} = \frac{\mu \beta }{i\omega \rho l}p_{0m} (b)\frac{{I_{1} \left( {\beta R} \right)}}{{I_{0} \left( {\beta R} \right)}}e^{i\omega t}$$

where *m* = 1, 2 are for case I and II, respectively.

The approximate transverse isotropic poroelastic constants for osteon and some necessary geometrical constants are grouped in Table 1. We select 0.04–0.3% [1, 33–35] as the strain range to investigate its relationship to the canalicular fluid velocity, pressure, flow rates, and shear stress. A physiological frequency range of 1–20 Hz [27] was selected to study the role of loading frequency. The canalicular radius ranged from 0.1 to 1 μm [38, 39].

**Table 1:** Geometrical and material characteristics used in the model [36]

*E*_*r*_ (GPa)	*μ*_*r*_	*E*_*z*_ (GPa)	*μ*_*z*_	M (GPa)	*a* (μm)	*b* (μm)
15.9	0.328	20.3	0.25	38	50	150

α	α′	*k* (m^2^)	*μ* (Pas)	*R* (m)	*ρ* (Kg m^−3^)	
0.132	0.092	10^−18^	10^−3^	1–10× 10^−7^	1000	

## Results

### Role of loading factor: axial strain amplitude ε_*z*0_ and frequency

**Fig. 3:**
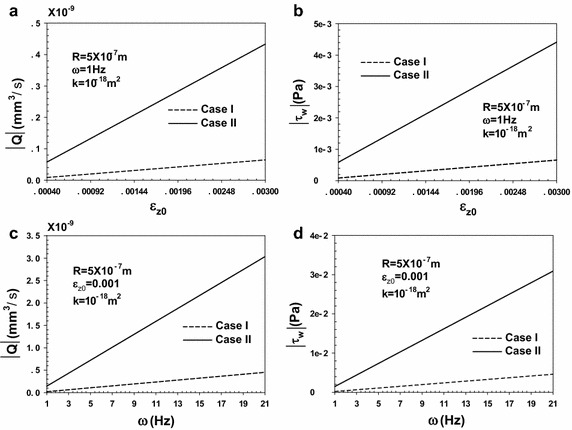
Fluid flow rate amplitude ($\left| Q \right|$, FFRA) and fluid shear stress amplitude ($\left| \tau_w \right|$, FSSA) as a function of the loading amplitude (*ε*_*z*0_) and frequency (*ω*) at *R* = 5 × 10^−7^ m and k = 10^-18^ m^2^. **a** Fluid flow rate amplitude as a function of the loading amplitude. **b** Fluid shear stress amplitude as a function of the loading amplitude. **c** Fluid flow rate amplitude as a function of the frequency. **d** Fluid shear stress amplitude as a function of the frequency

As shown in Fig. 3, the FFR amplitudes ($\left| Q \right|$, FFRAs) and FSS amplitudes ($\left| {\tau_{w} } \right|$, FSSAs) of the two cases are proportional to the loading amplitude (*ε*_*z*0_) and frequency (*ω*). When one of the two parameters (*ε*_*z*0_ or *ω*) is fixed, both the FFRA and FSSA have a linear relationship with the other parameter. Both the FFRA and FSSA produced in case II are also larger than those of case I.

### Dominating role: the strain rate

The amplitude of the strain rate, $\dot{\varepsilon }_{z}$, can be defined by the relationship of $\dot{\varepsilon }_{z} { = }\left| {i\omega \varepsilon_{z0} e^{i\omega t} } \right|{ = }\varepsilon_{z0} \omega$ [1, 18–22, 33–35] and flowing formulas for each case are obtained according to Eqs. (8), (9):

10 $$\left| {Q_{m} } \right| = \left| {\frac{2\pi }{{\omega^{2} \rho l}}p_{0m}^{*} (b)\left[ {\frac{R}{\beta }\frac{{I_{1} \left( {\beta R} \right)}}{{I_{0} \left( {\beta R} \right)}} - \frac{{R^{2} }}{2}} \right]} \right|\dot{\varepsilon }_{z} ;\quad m = 1,2 ,$$

11 $$\left| {\tau_{wm} } \right| = \left| {\frac{\mu \beta }{{\omega^{2} \rho l}}p_{0m}^{*} (b)\frac{{I_{1} \left( {\beta R} \right)}}{{I_{0} \left( {\beta R} \right)}}} \right|\dot{\varepsilon }_{z} ;\,\,\,\,\,m = 1,2 ,$$

where $p_{0m}^{*} (b) = p_{0m} {{(b)} \mathord{\left/ {\vphantom {{(b)} {\varepsilon_{z0} }}} \right. \kern-0pt} {\varepsilon_{z0} }}$,$p_{0m}^{*} (b) = p_{0m} {{(b)} \mathord{\left/ {\vphantom {{(b)} {\varepsilon_{z0} }}} \right. \kern-0pt} {\varepsilon_{z0} }}$. As shown in formulas (21)–(22), the FFRAs and FSSAs of the two cases are proportional to the amplitude of strain rate ($\dot{\varepsilon }_{z}$).

**Fig. 4:**
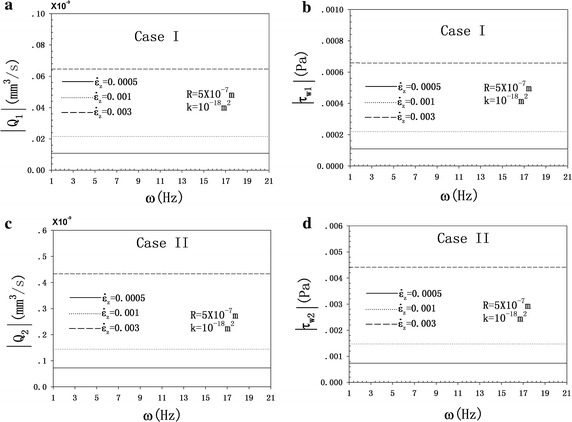
FFRA and FSSA as a function of the loading frequency at *R* = 5 × 10^−7^ m, *k* = 10^-18^ m^2^ and with the strain rate fixed at $\dot{\varepsilon }_{z}$ = 0.0005, 0.001, and 0.003 s^−1^. **a** FFRA as a function of the frequency for case I. **b** FSSA as a function of the frequency for case I. **c** FFRA as a function of the frequency for case II. **d** FFSA as a function of the frequency for case II

As shown in Fig. 4, the FFRAs and FSSAs of the two cases change little with the increase in frequency from 1 to 21 Hz with the strain rate fixed. Both the FFRA and FSSA depend more on the strain rate than on the loading frequency in a physiological loading state. Hence, the key role governing the fluid flow behavior and the FSS is the strain rate.

### Time responses of FFR and FSS

**Fig. 5:**
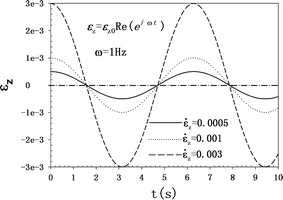
History of strain loads at ω = 1Hz and $\dot{\varepsilon }_{z}$ = 0.0005, 0.001, and 0.003 s^−1^, the operator *Re* () gives the real part of the complex number

**Fig. 6:**
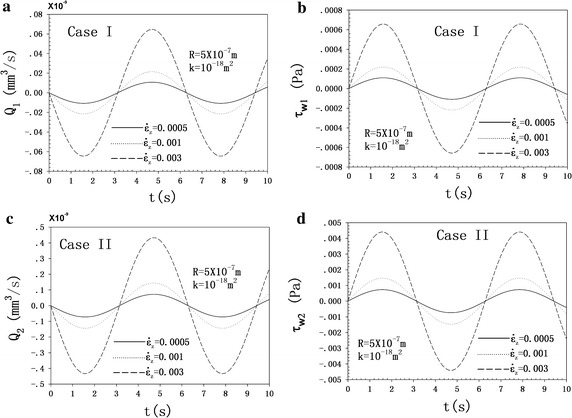
Time responses of FFR (**a** Case I; **c** Case II) and FSS (**b** Case I; **d** Case II) at *R* = 5 × 10^−7^ m, *k* = 10^-18^ m^2^

Figure 5 demonstrates the evolution of strain load with time. Based on Fig. 6, the evolutions of FFR and FSS with time are illustrated in Fig. 5. With the cyclic load, the induced FFR and FSS in the canaliculi are also cyclic, as shown in Fig. 6. Both the FFR and FSS in case II are larger (approximately 6.7 times) than those of case I.

### Effects of canalicular radius

**Fig. 7:**
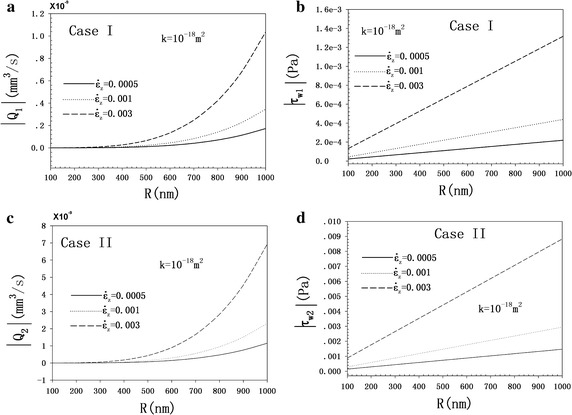
Evolutions of FFRA (**a**, **c**) and FSSA (**b**, **d**) with canalicular radius at *k* = 10^-18^ m^2^

Figure 7 illustrates the evolution of FFRA and FSSA with the canalicular radius. As shown in Fig. 7a and c, the FFRAs of both cases have a nonlinear increasing relationship with the canaliculus radius. The FSSAs of the two cases are proportional to the canalicular radius.

**Fig. 8:**
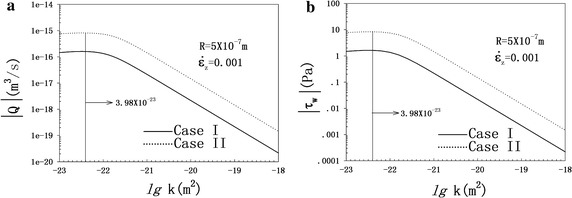
Effects of permeability with parameter values of *R* = 1 × 10^−7^ m, $\dot{\varepsilon }_{0} = 0.001$ s^−1^. Case I (*solid line*); case II (*dotted line*). **a** FFRA as a function of the permeability. **b** FSSA as a function of the permeability

### Role of permeability

The role of permeability is examined in Fig. 8 and plotted with the strain loading rate fixed at $\dot{\varepsilon }_{z} = 0.001\;s^{ - 1}$. According to [19] and [40], this parameter lies between 10^−23^ and 10^−18^ m^2^. We chose 10^−18^ m^2^ as a reference case to obtain the above results. The FFRAs and FSSAs of both cases experience a monotonic decrease with permeability from 10^−23^ to 10^−18^ m^2^, as shown in Fig. 8.

## Discussion

We present a theoretical model to describe the fluid flow and shear stress in the canaliculi of an osteon under axial harmonic loading. The current model links the mechanical loading at the osteon scale to the scale of canalicular fluid pressure, velocity, flow rates, and shear stress, which may have a significant stimulus to bone mechanotransduction.

We have developed the analytical solutions of the FFR and FSS as theoretical basis, and we aim to see the effects of the variation in boundary conditions and material parameters on the FFR and FSS. Loading boundary and the material parameters are the two major groups of factors that influence the FFR and FSS induced in the canaliculi. The linear increase of loading factors—strain amplitude and frequency—leads to the linear increase of FFRA and FSSA (see Fig. 4). However, the strain rate is the real loading factor for governing the canalicular fluid flow behavior (see Fig. 5). Although the actual fluid flow rates have not been measured in vivo, in vivo bone strains have been directly measured in humans (1200–1900 με [39]) during vigorous physical activities. The selected strain range of 0.04–0.3% cannot provide enough FSS (0.2–6 Pa, [23, 39]) for the cell to respond. However, some studies [41–43] indicate that a threshold of between 5 and 10% strain may generate this response. Therefore, the strain amplitude should be increased in the present investigation. A physiological frequency range of 1–20 Hz [38] is selected to examine its effect on FFR and FSS. The frequency has a substitutable role as the strain amplitude does on the FFR and FSS. This phenomenon could explain why Fritton et al. [44] observed that low-magnitude high-frequency mechanical loading appeared to have the same effect on maintaining bone mass. Live bones experience different physiological loading amplitudes for different frequencies, and loading amplitude has been reported to decrease as the frequency increases [44]. The loading amplitude and frequency are thus coupled in a physiological state. Therefore, the defined parameter, *ε*_z0_∙*ω* (strain rate amplitude), is reasonable and crucial, and it can be considered a representative loading parameter under a physiological state.

Canalicular fluid flow behavior depends not only on loading conditions, but also on geometric characteristics and material parameters. Figure 8 shows that the FFRAs and FSSAs increase as the canalicular radius increases. A large canalicular radius means the cross section where mass flux flows through is large, thereby increasing the FFRA. Permeability can be regarded as the macroscopic indicator of fluid flow at the microscopic level [19]. Published studies on determining the value of *k* mainly differ in terms of the bone scale. For heterogeneous structures, the local permeability value is related to a particular location on the osteon sample, and several experiments are required to obtain a representative or average value [45]. We selected the value 10^−18^ m^2^ as a reference case for the osteon associated to microscopic lacuno–canalicular.

The boundary conditions of case I allow fluid passage from the inner osteon wall and none across the outer elastic constrained wall. It can be assumed that the environmental liquid around the osteon can automatically produce physiological pressure on the cement surface to balance the pore pressure [33]. This boundary condition suggests that pore pressure is equal to the pressure of physiological liquid around the osteon. In case II, the fluid can freely passage from the inner wall but none across the outer wall, which is almost impossible or unrealistic for the osteon, while, it might be applicable and helpful for geomechanic engineering problems. In this case, the osteon cement surface is supposed to be perfectly rigid without fluid flowing through this surface (impermeable). This displacement boundary is an essential condition to obtain the analytical solutions. This assumption, which has been used in previous studies, may be rough and strong [18–23]. Superficially, case I seems closer to the physiological state than case II. Besides, as shown in Fig. 7, both the FFR and FSS induced in case II are larger (approximately 6.7 times) than those of case I.

In the model of Literature [6], the porous matrix medium is treated as isotropic material, while ours is transversely isotropic (osteon). They describe the fluid annulus surrounding the osteocytic process by a Brinkman equation for a fiber filled medium, while in our model its Navier–Stokes momentum equation. In our model, we disregarded the effects of fluid exchange in the lacuna and fiber matrix and focus on the first physical effects of canalicular fluid transport behavior—shear stress. Though their model seems more accurate, it is complicated and do not linking of cyclic mechanical loading on osteon scale to the fluid flow in the canaliculi scale. There are some unaddressed controversies in the view of morphology and anatomy. Such as theory must be a osteocyte processes fiber lays in the canaliculi and how many fibers each canaliculi contains? These issues have a large extent affecting the modelling method.

Strain-derived interstitial fluid stimuli, such as FSS [6] and fluid pressure gradient [46], serve an important function in bone mechanotransduction. Some other phenomena, such as piezoelectricity [47, 48], streaming potential [36, 49, 50], and chemotransport [51] in the PLC have also been postulated as mechanotransduction mechanisms for positive bone adaptation. Hence, further works, such as calculating these coupled physical effects generalized in the canaliculi and their role in cell response, need to be conducted.

The Hierarchical modeling processes have several limitations: (1) The proposed osteon boundary cases are the idealization of physiological conditions. (2) Every canaliculus is assumed to straightly run across the osteon wall and neglect the effect of lacuna. (3) A homogenized distribution of the canaliculus leads the permeability to be a constant. (4) One-dimensional (only longitudinal velocity component) canalicular fluid flow is considered.

## Conclusions

This hierarchical model firstly provides the analytical estimate of canalicular fluid flows in an axial loaded osteon. At the canaliculus scale, the fluid flow is described by a Stokes equation and its directly stimuli effect—FSS is focused on. This approach will enhance the evaluation of canalicular fluid flow related to bone mechanotransduction, and could also potentially account for other geological engineering problems. Despite the limitations of this study, the following conclusions are drawn:

I. The amplitudes of FFR and FSS are proportional to the strain amplitude and frequency. However, the key loading factor governing the FFR and FSS is the strain rate and it can be considered as a representative loading parameter in a physiological state.
II. The larger canalicular radius is, the larger FFRA and FSSA generalized, and the FSSA is proportional to the canalicular radius.
III. At the osteon scale, both the FFRAs and FSSAs experience a monotonic decrease as permeability increases.
IV. Both the FFR and FSS produced in case II (displacement confined osteon outer wall) are larger (approximately 6.7 times) than those of case I (elastic restrained osteon outer wall).

## Abbreviations

FSS: fluid shear stress
FFR: fluid flow rate
PLC: lacunar–canalicular porosity
FFRA: fluid shear stress amplitude
FSSA: fluid flow rate amplitude
FFRAs: fluid shear stress amplitudes
FSSAs: fluid flow rate amplitudes
